# To burn or not to burn: Comparing reintroducing fire with cutting an encroaching conifer for conservation of an imperiled shrub‐steppe

**DOI:** 10.1002/ece3.5461

**Published:** 2019-07-28

**Authors:** Kirk W. Davies, Roxanne C. Rios, Jon D. Bates, Dustin D. Johnson, Jay Kerby, Chad S. Boyd

**Affiliations:** ^1^ USDA – Agricultural Research Service Burns Oregon; ^2^ Oregon State University Burns Oregon; ^3^ The Nature Conservancy Burns Oregon

**Keywords:** ecological memory, exotic annual grass, fire surrogate, juniper, sagebrush, woody plant encroachment

## Abstract

Woody vegetation has increased on rangelands worldwide for the past 100–200 years, often because of reduced fire frequency. However, there is a general aversion to reintroducing fire, and therefore, fire surrogates are often used in its place to reverse woody plant encroachment. Determining the conservation effectiveness of reintroducing fire compared with fire surrogates over different time scales is needed to improve conservation efforts. We evaluated the conservation effectiveness of reintroducing fire with a fire surrogate (cutting) applied over the last ~30 years to control juniper (*Juniperus occidentalis* Hook.) encroachment on 77 sagebrush‐steppe sites. Critical to conservation of this imperiled ecosystem is to limit juniper, not encourage exotic annual grasses, and promote sagebrush dominance of the overstory. Reintroducing fire was more effective than cutting at reducing juniper abundance and extending the period of time that juniper was not dominating the plant community. Sagebrush was reduced more with burning than cutting. Sagebrush, however, was predicted to be a substantial component of the overstory longer in burned than cut areas because of more effective juniper control. Variation in exotic annual grass cover was explained by environmental variables and perennial grass abundance, but not treatment, with annual grasses being problematic on hotter and drier sites with less perennial grass. This suggests that ecological memory varies along an environmental gradient. Reintroducing fire was more effective than cutting at conserving sagebrush‐steppe encroached by juniper over extended time frames; however, cutting was more effective for short‐term conservation. This suggests fire and fire surrogates both have critical roles in conservation of imperiled ecosystems.

## 1 INTRODUCTION

Woody vegetation has increased substantially on rangelands worldwide over the past 100–200 years, causing ecosystem level land cover change that dramatically alters ecosystem function and can severely alter ecosystems services (Anadón, Sala, Turner, & Bennet, 2014; Archer et al., 2017; Huxman et al., 2005). Woody plant encroachment can degrade habitat for species of conservation concern, increase erosion and runoff risk, decrease biodiversity, and impair commodity production (Archer et al., 2011; Fulbright, Davies, & Archer, 2018; Miller, Svejcar, & Rose, 2000; Pierson, Bates, Svejcar, & Hardegree, 2007). Increases in woody vegetation have been attributed to numerous, often interacting factors, including decreased fire frequency, increased atmospheric CO_2_, altered grazing/browse regimes, and climate change (Archer, 1994; Bond & Midgley, 2000; D'Odorico, Okin, & Bestelmeyer, 2012). Arguably, increased atmospheric CO_2_ and decreased fire frequency from fire suppression and fine fuel reduction with grazing are the primary drivers of woody plant increases in many rangelands (Bond, 2008; Browning & Archer, 2011; Miller & Wigand, 1994). Grazing can reduce the likelihood of wildfire ignition and spread by removing fine fuel and increasing fuel moisture content (Davies, Boyd, Bates, & Hulet, 2016; Davies, Gearhart, Boyd, & Bates, 2017). Prolonged fire return intervals allow fire sensitive woody species to expand from fire safe areas into more productive areas and infill in areas where they historically were maintained at low densities (Briggs, Hoch, & Johnson, 2002; Johnson & Miller, 2008; Miller & Rose, 1995).

Reversing the trend of increasing woody dominance is often a goal of management for economic and ecological objectives. Management of woody vegetation is necessary to meet conservation goals of sustaining habitat for grassland, shrub‐steppe, and savannah obligate wildlife species (Archer et al., 2011; Fulbright et al., 2018). Reversing woody plant encroachment can be accomplished by reintroducing fire or using fire surrogates (Archer et al., 2011). However, conservation effectiveness (maintaining desired plant community) of reintroducing fire compared with fire surrogates at different time scales, especially over long time frames, is largely unknown.

There is a clear need to restore fire as a fundamental process to many ecosystems as this was often the historic disturbance limiting woody vegetation (Davis et al., 2000; Fuhlendorf, Woodward, Leslie, & Shackford, 2002). Thus, prescribed fire may be selected to control woody vegetation, especially at large spatial scales, as mimicking historic disturbance regimes is often assumed the best management for restoring ecosystems (Baker, 1994; Cissel, Swanson, & Wiesberg, 1999; Moritz & Odion, 2004; Suding, Gross, & Houseman, 2004). However, restoring historic disturbance regimes may not achieve conservation objectives as exotic plants, climate change, and other factors may have altered the response of the plant community to the historic disturbance regime (Davies, Svejcar, & Bates, 2009; Thorpe & Stanley, 2011). Long‐term absence of fire may also alter fuel, and reintroducing fire in these systems may have novel effects (Varner, Gordon, Putz, & Hiers, 2005), such as shifting the community to exotic dominated. Hotter and drier areas may also have lower resilience and resistance and be at increased risk of exotic plant invasion after fire (Chambers, Bradley, et al., 2014; Chambers, Miller, et al., 2014). Thus, there are concerns about the applicability of reintroducing fire in altered ecosystems.

Even if fire historically prevented woody plant encroachment, it may also not be selected because of restoration practitioners' and the public's aversion to using fire (Valkó, Török, Deák, & Tóthmérész, 2014). Fire can be challenging to contain, can only be applied under specific conditions, poses risk to property and life, and can cause short‐term decreases in livestock forage and wildlife habitat (Boyd et al., 2017; Lohmann, Tietjen, Blaum, Joubert, & Jeltsch, 2014; Valkó et al., 2014). Fire may also not be selected because of the risk of increasing exotic plants, which can dominate the seed bank and benefit more from fire than native plants (Stanley, Kaye, & Dunwiddie, 2011). Fire surrogates, such as cutting, are often selected over fire because they pose minimal risk, are easy to control, and can be applied across a broad range of conditions. This has led to some contradictory trends in conservation. For example, the USDA‐National Resource Conservation Service (NRCS) spends only 1% of conservation expenditures on fire, but two‐thirds of its expenditures on brush management, largely mechanical treatments, even though the NRCS recognizes the role of fire in conservation of these ecosystems (Twidwell, Allred, & Fuhlendorf, 2013). Conservationist and restoration practitioners, therefore, need to know the effects of fire compared with fire surrogates over extended time frames (multiple decades) to make informed decisions when selecting treatments to reverse woody plant encroachment to conserve imperiled ecosystems.

A concern with applying disturbances in any ecosystem is that ecological memory, which is maintained by biotic and abiotic legacies, may have been depleted (Blackhall et al., 2017; Johnstone et al., 2016). Climate change, invasive species, and community compositional changes from woody plant encroachment can diminish or even eliminate these legacies, resulting in a depleted ecological memory that is realized after the ecosystem is disturbed (Johnstone et al., 2016). Ecological memory is depleted when the combination of abiotic and biotic factors necessary for recovery after disturbance no longer exist. In contrast, resilience and resistance models are based on abiotic characteristics and do not account for biotic factors (Chambers, Bradley, et al., 2014; Chambers, Miller, et al., 2014). Disturbances in ecosystems with a depleted ecological memory can create novel ecosystem states (Williams & Jackson, 2007) that are maintained by a new set of biotic and abiotic legacies and reinforcing feedbacks (Bowman, Perry, & Marston, 2015; Scheffer, Carpenter, Foley, Folke, & Walker, 2001). Determining whether ecological memory remains is critical in woody plant‐encroached communities to limit unintended consequences of management and prepare for nonanthropogenic disturbances. Furthermore, determining whether ecological memory differs when reintroducing fire compared with a fire surrogate is needed to guide conservation efforts.

To determine whether the conservation effectiveness of reintroducing fire compared with a fire surrogate varies at different time scales (e.g., short‐ vs. long‐term post‐treatment) and whether ecological memory differs between reintroducing fire and applying a fire surrogate, we investigated treatments applied to conserve sagebrush (*Artemisia* L.) communities encroached by western juniper (*Juniperus occidentalis* Hook.). Western juniper, a fire‐intolerant shrub–tree, has expanded from 0.3 to 3.5 million ha since the late 19th century in the northern Great Basin and Columbia Plateau of the US, largely into sagebrush (*Artemisia* L.) dominated shrub‐steppe (Johnson & Miller, 2006; Miller, Bates, Svejcar, Pierson, & Eddleman, 2005; Miller et al., 2000), an ecosystem of conservation concern (Davies et al., 2011). Juniper has expanded largely because of elongated fire return intervals associated with fire suppression and historic overgrazing (Miller & Rose, 1995, 1999), and as juniper cover increases, erosion risk increases, sagebrush and herbaceous vegetation decrease, and habitat for sagebrush–obligate wildlife species is lost (Miller et al., 2005, 2000; Pierson et al., 2007). Prescribed fire and cutting are two commonly applied treatments to control juniper encroachment to restore sagebrush‐steppe communities (Miller et al., 2005). Recently, the use of fire to reverse western juniper expansion has come under scrutiny because fire is listed as a driving factor of the decline of sage‐grouse, a bird of conservation concern, and other sagebrush–obligate species (Boyd et al., 2017; USFWS, 2013) and concern over postfire invasion by exotic annual grasses (Condon, Weisberg, & Chambers, 2011). Risk of postfire exotic annual grass dominance is even greater if perennial grasses are reduced (Chambers, Roundy, Blank, Meyer, & Whittaker, 2007) as their resource use acquisition patterns overlap greatly with annual grasses (James, Davies, Sheley, & Aanderud, 2008). Maintaining perennial grasses to limit exotic annual grasses is a critical component of ecological memory in this ecosystem as exotic annual grass dominance can result in a new stable state that is maintained by an annual grass–fire cycle. Fire results in the temporary loss of sagebrush, which is undesirable for sagebrush–obligate wildlife species(Nelle, Reese, & Connelly, 2000). However, fire is speculated to result in longer juniper control than cutting (Miller et al., 2005) and, thus, fire may be more beneficial than cutting to sagebrush–obligate species over the long‐term if it does not promote increases in exotic annual grasses (Boyd et al., 2017). Therefore, it would be valuable to determine the conservation efficiency of using fire compared with cutting to control juniper across extended time frames. Critical to conservation of this ecosystem is to limit juniper, not promote exotic annual grass dominance, and encourage sagebrush dominance of the overstory.

The purpose of this study was to investigate the effects of reintroducing the historic disturbance (fire) compared with using a fire surrogate (cutting) for conservation of an imperiled shrub‐steppe being encroached by woody vegetation. We accomplished this task by sampling numerous cuts and burns that were applied over the last ~30 years to control juniper encroachment in sagebrush‐steppe communities. We hypothesized that the conservation effectiveness (i.e., community would have a sagebrush overstory) of reintroducing fire compared with cutting would vary over time and that ecological memory would be more depleted with reintroducing fire than cutting (i.e., exotic annual grass cover would be greater with burning). We also expected that exotic annual grass cover would be correlated with time since treatment, site environmental characteristics, and perennial grass abundance.

## 2 MATERIALS AND METHODS

### 2.1 Study area

The study was conducted in the northern Great Basin and Columbia Plateau in the northwest United States in mountain big sagebrush (*Artemisia tridentata* subsp. *vaseyana* (Rydb.) Beetle) communities that had been encroached by western juniper prior to the treatment. Areas sampled had either been cut or burned to control encroaching juniper, but not retreated or burned in a wildfire since initial treatment. Treatment areas sampled included public and private lands. Climate in this region is cool, wet winters, and hot, dry summers. Elevation of study sites ranged from 894 to 1,996 m above sea level with slopes from 0% to 50% and included all aspects. Common perennial grasses included Idaho fescue (*Festuca idahoensis* Elmer), bluebunch wheatgrass (*Pseudoroegneria spicata* (Pursh) A. Löve), bottlebrush squirreltail (*Elymus elymoides* (Raf.) Swezey), Thurbers needlegrass (*Achnatherum thurberianum* (Piper) Barkworth), Sandberg bluegrass (*Poa secunda* J. Presl), and western needlegrass (*Stipa occidentalis* Thurb. Ex S. Wats.). Common exotic annual grasses included cheatgrass (*Bromus tectorum* L.), Japanese brome (*Bromus japonicus* Thunb. Ex Murr.), and medusahead wildrye (*Taeniatherum caput‐medusae* (L.) Nevski). Native ungulates, other wildlife, and cattle occupied the study area and were not permanently excluded from the study sites. It is likely that cattle were excluded the first year or two after juniper control as this is a common management practice, but this information was frequently not available for the study sites.

### 2.2 Site selection and measurements

Potential study locations were identified using the Land Treatment Digital Library (USGS, 2018) and our knowledge of treatments on private lands. We identified 302 prescribed burn and 146 cut treatments sites. We evaluated all sites to determine whether they met criteria for inclusion in our study. Our criteria for inclusion were as follows: (a) the encroached plant community was a mountain big sagebrush community, (b) no pretreatments occurred (e.g., herbicide application), (c) no subsequent treatment or substantial disturbance after the initial control treatment (e.g., retreatment, wildfire, and seeding), (d) large enough to place our sample plot in with a 50 m buffer around it, (e) on a uniform slope and aspect, and (f) burned treatments were broadcast prescribed burns (i.e., not burning individual trees or small patches). This resulted in 47 burned and 30 cut sites, ranging in age from 3 to 33 years old, that met these criteria, and all were included in the study. Potential cut areas were most frequently excluded because they also included another treatment (burn, herbicide, seeding) or were retreated. Potential burned areas were largely excluded because they lack evidence of prefire juniper encroachment or were not a mountain big sagebrush community.

Vegetation characteristics at these sites were sampled in June or July of 2016 or 2017. Each site was sampled using a 100 × 100 m plot positioned ≥50 m away from treatment edges and roads. Vegetation was sampled along 5, 100‐m transects spaced 20 m apart within each plot. Juniper cover was determined using the line‐intercept method. Shrub, large perennial grass (perennial grass excluding Sandberg bluegrass), and exotic annual grass cover were measured using the line‐point intercept method with points every 2 m. Sandberg bluegrass was not included with the other perennial grasses because it is smaller in stature, develops phenologically earlier, and is less competitive with exotic annual grasses (Davies, 2008; James et al., 2008). Juniper density was measured by counting every tree rooted in 6 × 100‐m belt transects laid over each 100‐m transect. Shrub density was measured by counting every shrub rooted in 1 × 100‐m belt transects laid over each 100‐m transect. Density of large perennial grasses was measured by counting individuals rooted in 0.2/m^2^ quadrats place at 6 m intervals along each transect (16 quadrats per transect, 80 quadrats per 100 × 100 m plot).

Site environmental characteristics were sampled at the same time as vegetation characteristics. Slope was measured using a clinometer, and elevation was determined from digital elevation models and handheld Global Positioning Units. Aspect was measured with a compass and then converted to “northness.” Northness (*N*) was calculated by converting aspect to a continuous normalized variable where if aspect (*x*) was >180°, then *x* − 180° = *N*, and if *x* was less than 180°, then 180° − *x* = *N*. Woodland developmental phase at time of treatment was estimated from treatment project notes, and density and size of dead trees with developmental phases based on criteria from Miller et al. (2005). Phase I is characterized by sagebrush being the dominant overstory species with few juniper trees present. In Phase II, western juniper and sagebrush codominate the overstory. Phase III is when western juniper dominates the overstory, sagebrush is largely excluded from the community, and herbaceous production and diversity decreases. Time since treatment was determined by subtracting the treatment date from the sampling date.

### 2.3 Statistical analyses

*t* Tests (TIBCO Spotfire S+ ver. 8.2) were used to compare site characteristics and time since treatment between treatments. For these analyses, means were reported with standard error and were considered different at *p* < .05. We used PROC MIXED procedure in SAS (SAS ver. 9.4) to determine whether there was a treatment or time since treatment by treatment effect on vegetation response variables while accounting for site differences (slope, northness, elevation, and juniper developmental phase at treatment). Then, we used linear regression (SAS ver. 9.4) was used to evaluate juniper, sagebrush, other shrub (excluding sagebrush), and large perennial grass cover and density and annual grass cover relationship with time since treatment for each treatment. Other shrub category included bitterbrush (*Purshia tridentata* (Pursh) DC.), rabbitbrush spp. (*Chrysothamnus* Nutt. & *Ericameria* Nutt.), serviceberry (*Amelanchier utahensis* Koehne), horsebrush spp. (*Tetradymia* DC.), snowberry spp. (*Symphoricarpos* Duham.), mountain mahogany (*Cercocarpus ledifioius* Nutt.), Woods' rose (*Rosa woodsii* Lindl.), and currant spp. (*Ribes* L.). Data were evaluated using the SAS UNIVARIATE procedure to determine whether data met assumptions of regression analyses. Data that did not meet model assumptions were square‐root transformed to better meet model assumptions. The SAS REG procedure was used to fit simple linear regression models to each treatment's data, relating time since treatment to vegetation metrics; model fitness was evaluated by performing analysis of variance (ANOVA) *F* tests. If regression equations of both treatments were identified as significant for a response variable (i.e., treatment age was a reliable predictor), they were compared utilizing analysis of covariance (ANCOVA) with treatment age as the covariate. Regression coefficients were analyzed with the SAS GLM procedure and a MODEL statement that included the *X* variable, nominal variable, and interaction term (*H*_0_: *β*_1_ = *β*_2_). Specifically, it examined the effect of treatment type on the response variable while controlling for treatment age (3–33 years old). SAS GLM was also used to compare *y*‐intercepts by modifying the MODEL statement to exclude the interaction term. This assumed the slopes of the regressions lines were equal and tested the null hypothesis *α*_1_ = *α*_2_. Stepwise linear regression (TIBCO Spotfire S+ ver. 8.2) was used to evaluate the relationship between exotic annual grass cover and explanatory variables (treatment, northness, elevation, slope, perennial grass density, juniper development phase at treatment, and their interactions). Explanatory factors that were not significant contributors (as determined using stepwise selection at *α* = .05) were not included in the final model.

To estimate sagebrush and juniper cover at extended temporal ranges beyond the bounds of our study, we estimated the time that sagebrush was the dominant/codominant overstory species using regression models for sagebrush and juniper cover based on treatment and time since treatment (based on data from our study). Then, we modified our estimates based on the effect of juniper cover on sagebrush cover using Miller's model (Miller et al., 2000, p. 580; Figure 3) for juniper cover effects on sagebrush cover in mountain big sagebrush/Idaho fescue communities. Miller's model was developed from 49 sites, and juniper cover explained 92% (*R* ^2^ = .92) of variation in sagebrush cover (Miller et al., 2000). The rate of sagebrush cover decline in response to increasing juniper varies slightly by plant community (Miller et al., 2000). We used the model for mountain big sagebrush/Idaho fescue because it was developed from the most data and its estimates were between the other two models for wetter and drier communities. For simplicity, we considered sagebrush dominant/codominant in the overstory when its cover was ≥10%. To determine how long sagebrush cover was ≥10%, we used our regression models to predict sagebrush and juniper cover over time. We then used our estimated juniper cover to estimate sagebrush cover using Miller's model (Miller et al., 2000). Miller's model uses the effect of juniper cover on sagebrush to estimate sagebrush cover. We used sagebrush cover predicted by our models over time until Miller's model predicted lower sagebrush cover than our model based on juniper cover effect, and then, we used Miller's model predicted value for sagebrush cover. We also estimated sagebrush dominance/codominance in the burned areas using a more conservative method by determining the lag in juniper density between cut and burned areas and then assuming that that difference was the same for juniper cover.

## 3 RESULTS

Time since treatment application was not different between the burned (15.8 ± 1.2 years) and cut (14.2 ± 1.4 years) treatments (*p* = .399). Woodland development phase at the time of the treatment was similar between treatments (*p* = .323) with most areas being in Phase II at the time of treatment (Burn = 22%, 53%, and 24% and Cut = 27%, 63%, and 10% in Phase I, II, and III at time of treatment, respectively). Elevation was similar between burned (1,515 ± 36 m) and cut (1,548 ± 34 m) treatments (*p* =.514). Aspect (as northness) was similar between treatments (*p* =.125), but slope was greater in the cut (16 ± 2%) compared with the burned (10 ± 1%) treatment (*p* = .029).

**Figure 1:**
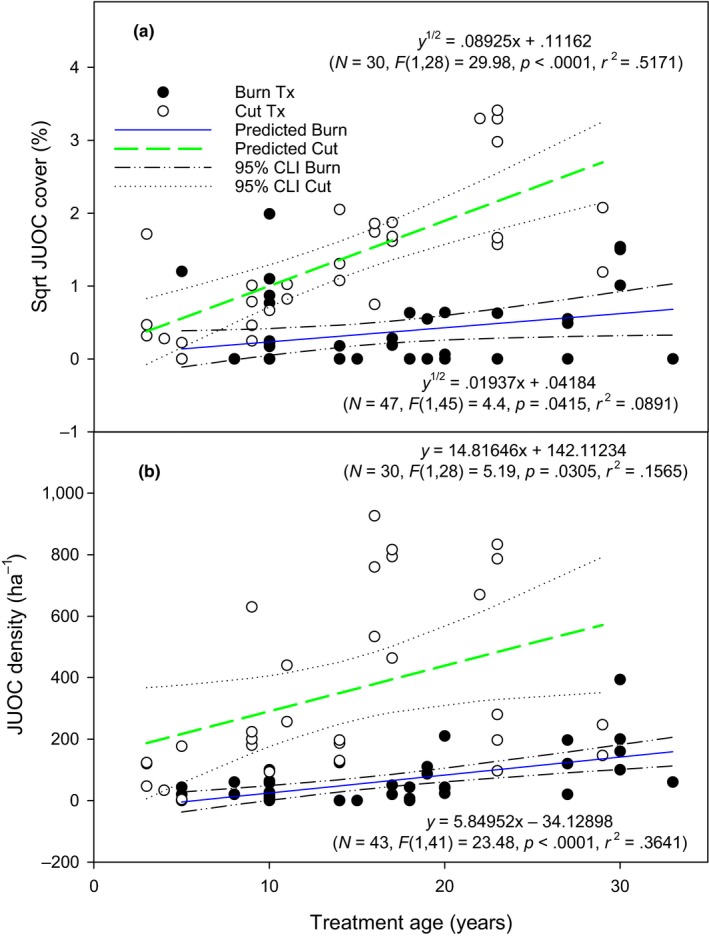
Square‐root transformed western juniper (Sqrt JUOC) cover (a) and juniper (JUOC) density (b) relationship (linear regression lines with 95% confidence intervals) with time since treatment in cut and burned areas. Top and bottom model refers to the cut and burn treatment, respectively

Juniper cover was influenced by the interaction between treatment and time since treatment (*p* < .001). Juniper cover increased with time since treatment in burned and cut areas (Figure 1a; *p* = .042 and <.001, respectively). Time since treatment explained 9% and 52% of the variation in juniper cover in the burned and cut treatments, respectively (*R* ^2^ = .089 and .517, respectively). Juniper cover increased at a faster rate in the cut compared with the burned treatment (*p* < .001), and burning decreased juniper cover more than cutting (*p* < .001). Thirty years after treatment, juniper cover was estimated to be 0.4% and 7.8% in the burned and cut treatments, respectively. Juniper density was not influenced by the interaction between treatment and time since treatment (*p* = .692), but differed between treatments (*p* = .009). Density of juniper increased with time since treatment in burned and cut areas (Figure 1b; *p* < .001 and .031, respectively). Time since treatment explained 36% and 16% of the variation in juniper density in burned (*R* ^2^ = .364) and cut *R* ^2^ = .157) areas. The rate of juniper density increase over time was similar between treatments (*p* = .107). Burning decreased juniper density more than cutting (*p* < .001). Estimated juniper density averaged 141 and 587 individuals per∙hectare 30 years after treatment in burned and cut areas, respectively.

**Figure 2:**
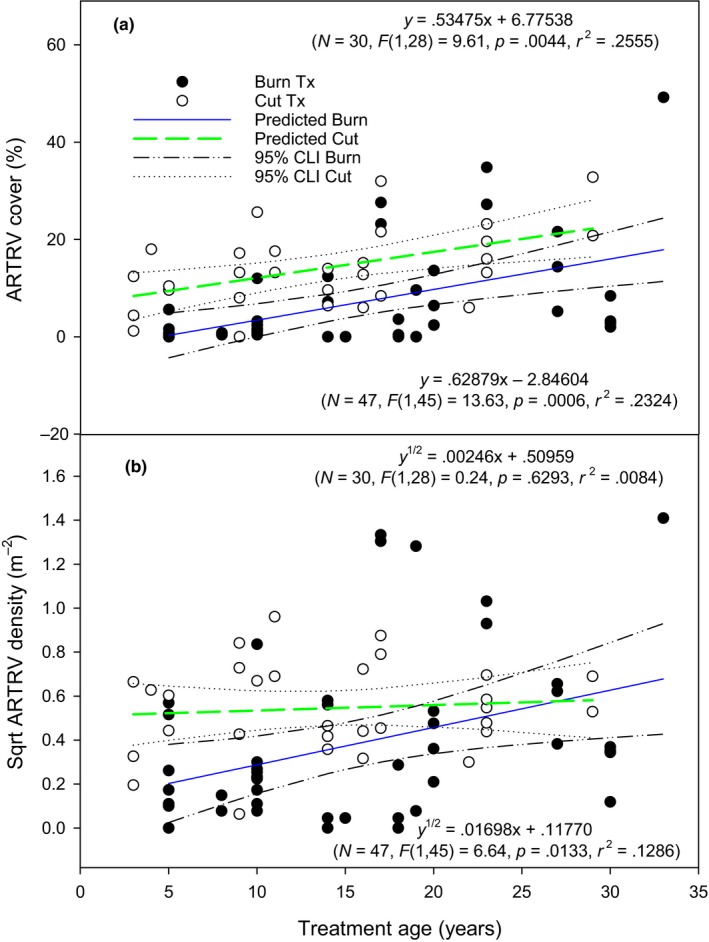
Mountain big sagebrush (ARTRV) cover (a) and square‐root transformed sagebrush (Sqrt ARTRV) density (b) relationship (linear regression lines with 95% confidence intervals) with time since treatment in cut and burned areas. Top and bottom model refers to the cut and burn treatment, respectively

The interaction between treatment and time since treatment influenced mountain big sagebrush cover (*p* = .029). Mountain big sagebrush cover increased with time since treatment in burned and cut areas (Figure 2a; *p* < .001 and .004, respectively). Time since treatment explained 23% and 25% of the variation in sagebrush cover in the burned (*R* ^2^ = .232) and cut (*R* ^2^ = .256) treatments. The post‐treatment rate of sagebrush cover increase did not differ between treatments (*p* = .718). However, burning resulted in less initial sagebrush cover compared with cutting (*p* < .001). Sagebrush density was influenced by the interaction between treatment and time since treatment (*p* = .021). Sagebrush density increased with time since treatment in the burned areas, but not in cut areas (Figure 2b; *p* = .013 and .629, respectively). Time since treatment explained 12% of the variation in sagebrush density in the burned areas (*R* ^2^ = .123).

**Figure 3:**
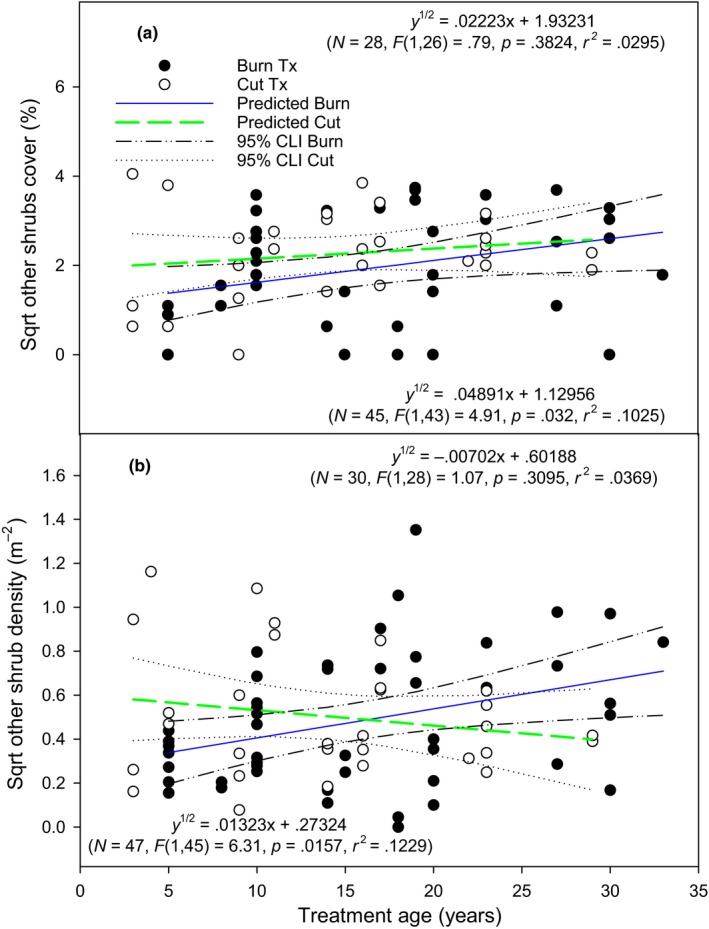
Square‐root transformed other shrub cover (a) and density (b) relationship (linear regression lines with 95% confidence intervals) with time since treatment in cut and burned areas. Other shrub is all shrubs except for mountain big sagebrush. Top and bottom model refers to the cut and burn treatment, respectively

Other shrub cover and density were influenced by the interaction between treatment and time since treatment (*p* = .012 and .002). In burned areas, cover of shrubs other than sagebrush increased with time since treatment, but not in cut areas (Figure 3a; *p* = .032 and .382, respectively). Other shrub density increased with time since treatment in burned areas (*p* = .016), but not in cut areas (Figure 3b; *p* = .310). Time since treatment explained 10% (*R* ^2^ = .103) and 12% (*R* ^2^ = .123) of the variation in other shrub cover and density in burned areas, respectively.

Perennial grass cover and density were similar between cut and burned areas and were not influenced by the interaction between treatment and times since treatment (*p* > .05). Perennial grass cover did not differ with time since treatment in either burned or cut areas (*p* = .442 and .518, respectively). Similarly, perennial grass density was not influenced by time since treatment in burned (*p* = .185) and cut (*p* = .548) areas. Treatment by time since treatment did not influence exotic annual grass cover (*p* = .487). Exotic annual grass cover was similar between the burned (17.5 ± 2.6%) and the cut (16.4 ± 2.6%) treatment (*p* = .759). Exotic annual grass cover did not differ with time since treatment in burned and cut areas (*p* = .886 and .065, respectively). Exotic annual grass cover was influenced by northness, elevation, large perennial grass density, and their interactions (Table 1; *p* < .001, Adjusted *R* ^2^ = .551). Exotic annual grass cover generally decreased as northness, elevation, and large perennial grass density increased. Treatment, woodland development phase at time of treatment, slope, and their interactions with each other and other variables did not explain exotic annual grass cover (*p* > .05).

**Table 1:** Final stepwise linear regression model of exotic annual grass cover relationship with explanatory variables in mountain big sagebrush communities where encroaching western juniper had been controlled with cutting or burning (Adjusted *R* ^2^ = .551)

Coefficient	Value	*SE*	*T*‐value	*p*‐Value
Intercept	242.83	44.53	5.45	<.001
PG density	−15.81	4.83	−3.27	.002
Northness (*N*)	−1.26	0.36	−3.45	.001
Elevation (Elev)	−0.13	0.03	−4.22	<.001
*N*:Elev	<0.01	<0.01	2.68	.009
*N*:PG density	0.09	0.03	2.65	.010
Elev:PG density	0.01	<0.01	2.89	.005
*N*:Elev:PG density	<0.01	<0.01	−2.40	.019

Note

PG density = large perennial grass density, Northness (*N*) was calculated by converting aspect to a continuous normalized variable where if aspect (*x*) was >180°, then *x *− 180° = *N*, and if *x* was less than 180°, then 180° − *x* = *N*.

**Figure 4:**
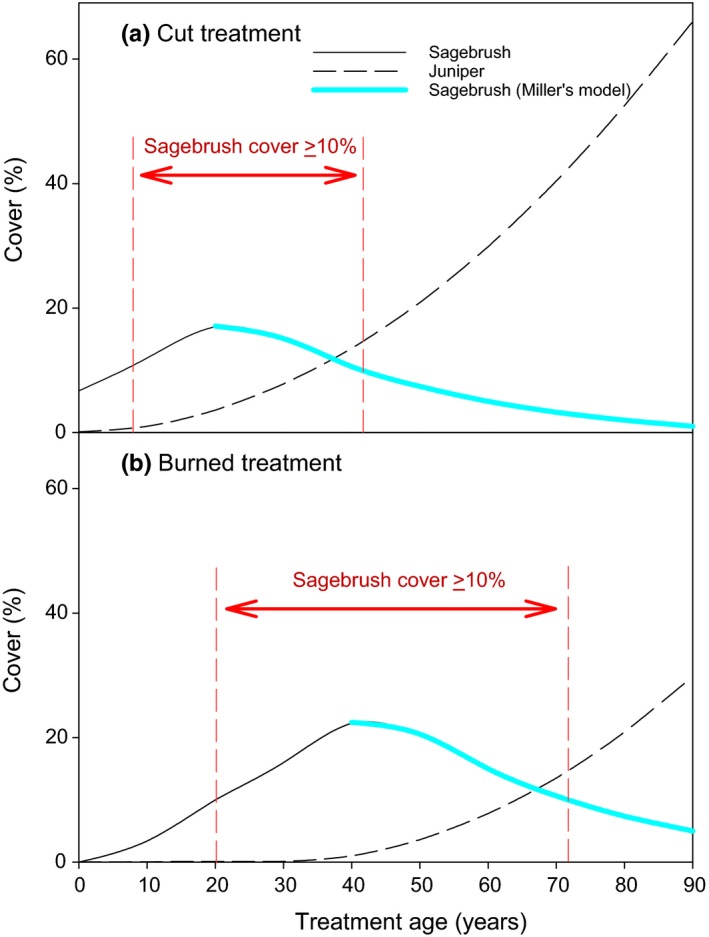
Estimated mountain big sagebrush cover after cutting (a) and burning (b) treatments applied to control juniper over 90 years post‐treatment adjusted for the estimated effects of juniper cover on sagebrush cover (blue line) using Miller's model (Miller et al., 2000). The burned treatment is a conservative estimate of sagebrush cover because we assume only a 30‐year lag in juniper cover between burned and cut treatments (based on an observed 30‐year lag in juniper density in burned compared with cut areas). Sagebrush and juniper cover were estimated using our regression models, and we then used our predicted juniper cover to estimate sagebrush cover using Miller's model (Miller et al., 2000) for Idaho fescue/mountain big sagebrush communities. We use sagebrush cover predicted by our models until Miller's model predicted lower sagebrush cover than our model based on juniper cover effect (blue line), and we then used Miller's model predicted value for sagebrush cover

Sagebrush was estimated to be dominant/codominant (≥10% cover) for 36 years in cut areas (Figure 4a) and 178 years in burned areas. Using a more conservative estimate (assuming only a 30‐year lag in juniper cover difference between cut and burned areas) for burned areas, sagebrush dominance/codominance was estimated to occur for 52 years (Figure 4b).

## 4 DISCUSSION

Reintroducing the historic disturbance (fire) resulted in longer control of encroaching western juniper compared with a fire‐surrogate (cutting) treatment. More juniper trees, mostly seedlings, survived the cutting treatment compared with the burn, resulting in an estimated 142 individuals per∙hectare in cut areas remaining after treatment. This is in contrast with results from forested ecosystems, where mechanical treatments reduced tree density more than burning (Schwilk et al., 2009). Burning likely also destroyed a large number of juniper seeds, but cutting juniper had no effect on juniper seeds (Miller et al., 2005). The combined effect of better juniper control and likely reduction of juniper seeds resulted in burned areas not reaching the initial density observed in the cut areas until 30 years after treatment, though a few trees in some of the sites survived burning. Thus, there was a 30‐year lag in juniper density between burned and cut areas. The much greater initial density of juniper after treatment in cut compared with burned areas explains why juniper cover increased substantially more rapidly in cut areas.

One of the major questions when selecting a conservation action for reversing woody plant encroachment is how long it takes before woody plants redominate after treatment; in other words, how long is the treatment effective. For this discussion, we are assuming that western juniper dominance occurs at approximately 25% cover, a level of cover that reduces sagebrush cover to <25% of its potential in wetter sites, and is representative of a closed juniper woodland in drier sites (Miller et al., 2005, 2000). Our regression models predict that it will take 257 and 55 years to reach juniper cover of 25% in burned and cut treatments, respectively. However, both time frames are outside of our data range and we expect that in burned areas, since junipers were largely absent immediately after treatment, juniper cover will, after a certain point, increase at a more rapid rate than in the first 30 years post‐treatment. If we assume the 30‐year lag in densities between cut and burned treatments applies to juniper cover, then 85 years is a conservative estimate of the time frame for juniper cover to reach 25% in the burned treatment. Our estimate is supported by fire history analyses that found that it takes 80 to >120 years to approach stand closure after wildfires in mesic and dry sites, respectively (Johnson & Miller, 2006).

Burning reduced mountain big sagebrush cover more than cutting, but sagebrush cover increased at a similar rate following treatment regardless of treatment type. However, the more rapid increase in juniper cover in cut areas over time will result in a decline in sagebrush cover sooner than in burned areas. Sagebrush cover declines exponentially as juniper cover increases (Miller et al., 2000). At 25% juniper cover, mountain big sagebrush cover was 0%–1% and <10% on drier and wetter sites, respectively (Miller et al., 2000). Based on the treatment‐dependent rate of post‐treatment juniper development, a conservative estimate of the difference between cut and burned areas suggests that sagebrush will dominate/codominate the overstory 44% longer on burned areas (Figure 4). Mountain big sagebrush cover, in general, can be rapidly recovered after fire in western juniper‐encroached shrub‐steppe with seeding sagebrush (Davies & Bates, 2017; Davies, Bates, & Boyd, 2019; Davies, Bates, Madsen, & Nafus, 2014). With successful seeding, sagebrush dominance can be extended to the majority of the time interval that juniper is controlled in burned areas.

A concern with woody plant treatments, including juniper removal in sagebrush ecosystems, is their potential to cause undesirable shifts in the plant community, in particular increased exotic plants (Archer et al., 2011; Bates, Sharp, & Davies, 2014; Bates, Svejcar, Miller, & Davies, 2017; Davies et al., 2019; Roundy, Miller, et al., 2014). Exotic annual grass increases, however, are generally assumed to be more problematic with burning, likely because of the positive feedback cycle between exotic annual grasses and fire (Chambers et al., 2007; D'Antonio & Vitousek, 1992; Rossiter, Setterfield, Douglas, & Hutley, 2003). Supporting this assumption, Williams et al. (2017) found in a short‐term study that exotic annual grass cover was greater in burned than cut treatments in sagebrush communities with moderate tree dominance. Surprisingly, our results did not support this assumption; rather, our results suggest that in some situations both treatments can have a post‐treatment exotic annual grass issue. Thus, ecological memory was independent of treatment applied to reverse woody plant encroachment. Ecological memory was largely related to environmental characteristics, with exotic annual grasses being more problematic on hotter, drier sites, similar to resilience and resistance models (Chambers, Bradley, et al., 2014; Chambers, Miller, et al., 2014). Similar to other studies (Chambers et al., 2007; Corbin & D'Antonio, 2004; Davies, 2008), we also found that exotic annual grass cover was correlated negatively to perennial grass density. This suggests that the density of perennial grasses as well as environmental characteristics can be used to estimate where ecological memory has been depleted, and thus, exotic annual grasses may be more problematic post‐treatment. These variables can be used to prioritize areas for conservation action and determine where additional treatments may be needed based on the likelihood of substantial exotic annual grass response. In juniper‐encroached shrub‐steppe, post‐treatment seeding of perennial vegetation can reduce the probability of substantial increases in exotic annual grasses (Davies et al., 2019). Additional treatments may need to be integrated with woody vegetation treatments in other areas where ecological memory is depleted to achieve conservation goals.

Though our results suggest that exotic annual grasses may be a post‐treatment risk in some areas, sooner or later these sites will likely burn in wildfires (Davies et al., 2011); therefore, treating juniper should not be abandoned because of risk of exotic plants increasing after treatment. Furthermore, allowing junipers (Bates et al., 2014; Miller et al., 2011; Miller, Tausch, MacArthur, Johnson, & Sanderson, 2008) and other woody species (Pierce, Meyer, & Jull, 2004; Twidwell, Rogers, et al., 2013) to continue to grow and infill results in increased fuel loads that can cause more severe wildfires. In altered ecosystems, postdisturbance increase in exotic plants is probably the new reality. Preemptively planning when these disturbances occur (i.e., prescribed burning) and having the resources and materials (e.g., seeds) available for additional postdisturbance treatments is probably more judicious than attempting to restore these communities after wildfires when resources and materials can be limited. Restoration practitioners and other resource managers need to be aware of the probability of post‐treatment exotic plant increases in woody‐encroached communities and plan accordingly to minimize this risk.

Mechanical treatments and burning to control conifer encroachment can increase exotic annual grasses because of increased soil nutrient and water availability (Bates et al., 2017; Roundy, Young, et al., 2014). The variability in exotic annual grass cover post‐treatment shown in our study has also been observed in several short‐term studies in western juniper and piñon (*Pinus* ssp.)‐juniper (*Juniperus* ssp.) encroached sagebrush communities (Bates et al., 2019, 2014, 2017; Chambers, Miller, et al., 2014; Roundy, Miller, et al., 2014). Some of the variability in exotic annual grass post‐treatment response has been attributed to differences in tree dominance, with exotic annual grasses being more problematic on more developed woodlands post‐treatment (Bates et al., 2014; Roundy, Miller, et al., 2014; Williams et al., 2017). Similar to our results, short‐term studies have also observed that exotic annual grass cover is generally greater on hotter and drier sites post‐treatment (Bates & Davies, 2017; Chambers, Bradley, et al., 2014; Chambers, Miller, et al., 2014; Roundy, Miller, et al., 2014). Our results demonstrate that this can be a persistent alteration of the composition of the plant community, not just a short‐term response to disturbance.

The greater abundance of cheatgrass in the hotter, drier sites after disturbance suggests that ecological memory has been depleted in these juniper‐encroached mountain big sagebrush communities, especially where perennial grass abundance has declined. Thus, where ecological memory has been lost, a novel ecosystem state may develop after disturbance (Johnstone et al., 2016), in this case an exotic annual grassland. This loss of ecological memory may potentially become an issue on even cooler and wetter sites as climate becomes hotter and drier in the summer (Mote & Salathé, 2010). Substantial exotic annual grass invasion will increase the probability of developing an annual grass–fire cycle which will create a novel annual grass state (D'Antonio & Vitousek, 1992). Novel states can be maintained by a new set of abiotic and biotic legacies and reinforcing feedbacks (Bowman et al., 2015; Scheffer et al., 2001). Two trajectories appear to be developing in juniper‐encroached mountain big sagebrush communities, one where ecological memory is being maintained in cooler and wetter sites and another where ecological memory is depleted in hotter and drier sites. Other plant communities that have broad ecological amplitude may similarly be developing divergent responses to disturbances based on variation in ecological memory along environmental gradients.

Reintroducing fire is a more effective treatment for long‐term conservation of sagebrush‐steppe than cutting; however, cutting was more effective for short‐term conservation because it did not reduce sagebrush. Burning is also generally less expensive than cutting (Boyd et al., 2017), and our results show it increases the time period that sagebrush is a substantial component of the plant community. Our results are counter to recommendations to limit fire in all sagebrush communities (USFWS, 2013) and a general aversion to using fire in many countries (Valkó et al., 2014). Reintroducing this historic disturbance is likely needed to restore these communities and as well as other woody plant‐encroached communities. However, there are situations where prescribed fire is not practical, especially near areas with expanding human populations and development. Cutting can be a surrogate for fire in these situations, but will have to be applied more frequently than fire to achieve similar long‐term results. Thus, a cutting treatment with a follow‐up cutting treatment may be a surrogate for burning. Cutting juniper may also be more in line with short‐term management goals, such as maintaining current sagebrush cover levels (Williams et al., 2017). Cutting and burning can also be part of an integrated management plan that maintains sagebrush dominance on a larger portion of the landscape across multiple temporal scales (Boyd et al., 2017), which can be critically important to wildlife species that depend on shrub‐steppe communities.

Sagebrush–juniper dynamics were historically regulated by periodic fire (Miller & Rose, 1995, 1999; Miller et al., 2000). The modern‐day presence of exotic annual grasses imparts new consequences to interruptions in the fire cycle in the form of a loss of ecological memory. Our results, however, demonstrate that reintroducing fire after decades of absence can achieve desired results in imperiled ecosystems even with an exotic plant threat. We do caution practitioners that ecological memory may be lost with juniper encroachment in hotter and drier sagebrush communities and that future climate change scenarios suggest this may expand into wetter, cooler sagebrush‐steppe, especially if this is coupled with a reduction in perennial grasses. Ecological memory has probably been depleted in other woody plant‐encroached communities and will likely become more extensive in areas that become hotter and drier with climate change.

## CONFLICT OF INTEREST

None declared.

## **AUTHORS**'** CONTRIBUTIONS**

CSB, KWD, JDB, JK, DDJ, and RCR conceived and designed the experiment; RCR and JDB collected the data; RCR and KWD analyzed the data; KWD led the writing of the manuscript; and all authors contributed to drafts of the manuscript and gave final approval for publication.

## Data Availability

Data available at: https://doi.org/10.5061/dryad.9rq4743
